# Paternalistic leadership and counterproductive work behavior: mediating role of leader identification and moderating effect of traditionality in Chinese generation Z employees

**DOI:** 10.3389/fpsyg.2025.1587525

**Published:** 2025-07-08

**Authors:** Yangjie Ke, Li Liu, Manli Gu

**Affiliations:** School of Business, Taylor's University, Subang Jaya, Malaysia

**Keywords:** paternalistic leadership, counterproductive work behavior, leader identification, Chinese traditionality, generation Z employees

## Abstract

**Introduction:**

This study investigates how paternalistic leadership (authoritarian, benevolent, and moral) affects counterproductive work behavior among Chinese Generation Z employees. By integrating culturally specific leadership styles with generational traits, this research offers insights into the mechanisms driving counterproductive work behavior in the Chinese workplace. Drawing upon social identity theory and the cultural context of traditional China, we propose a moderated mediation model where leader identification acts as a mediator and the employee’s traditionality serves as a moderator.

**Methods:**

We collected data through a multi-wave survey of 324 Gen Z employees in China. The collected data were analyzed in Stata 17.0 using multiple regression analysis, examining the relationship between paternalistic leadership and counterproductive work behavior.

**Results:**

Results reveal that benevolent and moral leadership reduce counterproductive work behavior, while authoritarian leadership increases it. Leader identification fully mediates the effects of benevolent and moral leadership, and partially mediates the effect of authoritarian leadership. Traditionality negatively moderates the relationship between paternalistic leadership and leader identification, with lower traditionality strengthening this connection.

**Discussion:**

These results highlight the complex dynamics between leadership styles and employee behavior, providing insights for creating productive and harmonious workplaces for Gen Z employees in China. The findings also emphasize leader identification as a key mechanism and traditionality as a contextual moderator shaping these effects, underscoring the need for adaptive leadership approaches.

## Introduction

Counterproductive work behaviors (CWB)—such as resistance to instructions, absenteeism, and interpersonal conflicts—have become a growing concern in China’s competitive and high-pressure workplace (Zhu and Zhang, 2021). These behaviors are especially concerning among Generation Z employees, who are rapidly becoming a major workforce segment (Fan et al., 2023). Leadership style plays a pivotal role in shaping employee attitudes and behavior (Walumbwa and Hartnell, 2011). Within the Chinese cultural and organizational context, paternalistic leadership is widespread in Chinese organizations and deeply rooted in Confucian values (Farh et al., 2008). This leadership style has been shown to shape various employee behaviors, including voice behavior (Peng and Chen, 2022), innovative work behavior (Nazir et al., 2020), and sustained work behavior (Fang et al., 2019). This raises a critical question: how does paternalistic leadership influence the counterproductive behaviors of Chinese Gen Z employees?

Although Gen Z employees across countries share traits such as creativity, confidence, and a strong sense of fairness (Dangmei and Singh, 2016; Nova et al., 2022), Chinese Gen Z employees display distinctive characteristics shaped by both generational and cultural contexts. Compared with previous generations in China, they are generally more individualistic, expressive, and sensitive to authority, having grown up during a time of rapid economic growth, technological advancement, and increasing global exposure (Hendrastomo and Januarti, 2023). At the same time, unlike their Western counterparts, Chinese Gen Z employees have been shaped by Confucian family values and a tradition of hierarchical relationships that emphasize respect for authority and social harmony (Zhang N. et al., 2021). In addition, they have grown up in a distinct social and cultural environment—particularly an internet landscape characterized by tighter governance and regulation—which may shape how they access information, form opinions, and interact with authority. For example, internet governance and social media restrictions in China have been shown to influence the behaviors and values of Generation Z (Xu and Albert, 2014). These generational and cultural differences may influence how Chinese Gen Z employees perceive and respond to leadership, particularly traditional forms such as paternalistic leadership.

While existing research has explored various leadership styles such as transformational (Huang et al., 2021), ethical (Shen and Lei, 2022), and exploitative (Guo et al., 2023), there is a gap in understanding how culturally leadership styles, like paternalistic leadership, affect counterproductive work behaviors among this demographic. To better understand this relationship, it is important to consider the psychological mechanisms and cultural factors that influence how employees respond to leadership. Drawing on social identity theory (Tajfel and Turner, 1979), we investigate how leader identification mediates the relationship between paternalistic leadership and counterproductive work behaviors (Wang and Howell, 2012). We further argue that traditionality—the degree to which individuals endorse hierarchical, Confucian values—moderates this relationship by influencing how strongly Gen Z employees identify with paternalistic leaders (Tan et al., 2021; Li et al., 2017; Li and Sun, 2015). This study seeks to address this gap by examining the mediating role of leader identification and the moderating role of traditionality in the context of paternalistic leadership.

Building on this conceptual framework, understanding how paternalistic leadership influences counterproductive work behavior among Chinese Gen Z employees holds both theoretical and practical significance. Theoretically, this research addresses a critical gap by examining how a culturally rooted leadership style—paternalistic leadership—affects the counterproductive behaviors of a new generational workforce. Specifically, by identifying leader identification as a mediator, the study clarifies how paternalistic leadership influences counterproductive work behavior. Furthermore, by examining traditionality as a generational trait, it shows how differences among Chinese Gen Z employees shape their responses to such leadership. Practically, the study provides evidence-based guidance for organizations to develop leadership strategies tailored to the characteristics of China’s evolving workforce. These insights support the design of leadership practices that are both culturally appropriate and effective in managing younger employees.

## Theoretical background and hypothesis development

### Theoretical background

Paternalistic leadership represents an indigenous aspect of Chinese leadership, fundamentally rooted in Confucian ideology (Westwood, 1997). This leadership style encompasses traits of fatherly kindness, moral integrity, stringent discipline, and authoritative guidance (Pellegrini and Scandura, 2008). Owing to cultural influences, paternalistic leadership finds wide adoption, particularly within family-run businesses in China (Farh and Cheng, 2000). Farh and Cheng provide a comprehensive framework for paternalistic leadership, defining it across three dimensions: authoritarianism, benevolence, and morality. Authoritarianism summarizes the leader’s exercise of absolute authority and the expectation of unwavering obedience. Benevolence, on the other hand, denotes a leader’s inclination toward personalized concern for the well-being of individuals beyond the confines of professional relationships. The moral dimension signifies a leader’s representation of personal virtue, self-discipline, and selflessness. Notably, empirical investigations have unveiled cross-dimensional correlations within paternalistic leadership, revealing positive associations between benevolence and morality while showing a negative linkage with authoritarianism (Pellegrini et al., 2007). The triad model of paternalistic leadership has gained widespread recognition and serves as the foundation for a multitude of subsequent research (Pellegrini and Scandura, 2008). While the term “paternalistic leadership” may carry different meanings in other cultural or theoretical contexts, sometimes even being associated with controlling or condescending leadership styles that limit employee autonomy (Aycan, 2006). This study adopts a culturally specific interpretation rooted in the Confucian tradition, as our research is situated in the Chinese context.

Counterproductive work behavior is defined as the deliberate actions of employees that either harm or represent a risk to the company and its stakeholders (Bolton et al., 2012). It is often referred to as workplace deviance and is characterized as “voluntary behavior that violates significant organizational norms and threatens the well-being of an organization or its members” (Gruys and Sackett, 2003). According to the stressor-emotion model, the counterproductive work behavior originates from stressful work situations, leading to negative emotions among employees (Spector and Fox, 2002). Leadership factors as a prevalent form of stress is a key element affecting the counterproductive work behavior (Holtz and Harold, 2013). For example, ethical leadership is negatively associated with employees’ counterproductive work behavior, and transactional leadership intensifies the connection between workplace stress and adverse employee behaviors (Yao et al., 2014). Within the exploration of factors contributing to counterproductive work behavior, one potential area of conflict in multigenerational workplaces is the field of management and leadership styles (Arsenault, 2004). In this particular context, the domain is notably impacted by differences between generations in regards to retention, values, motivation, work style preferences, and perceptions of effective leadership. The central conflict often revolves around what it means to be a leader and the attributes associated with being a good leader (Kraus, 2017).

One of the most influential theories explaining how individuals perceive themselves within organizations is social identity theory (Tajfel and Turner, 1979). This theory suggests that individuals categorize themselves and others into different social groups, enabling individuals to locate or define themselves in the social environment. Within this framework, leader identification takes place when employees positively evaluate both the role and personal identity of their leader (Sluss and Ashforth, 2008). Leader identification will encourage employees to align their perceptions of the leader with their own self-concept (Bakker et al., 2023), leading them to strive to meet the leader’s expectations and act in ways that benefit the leader (Johnson, 2010). Leader identification, which refers to how employees define themselves in their relationship with their leaders, has been considered a crucial psychological mechanism through which leadership styles influence employees’ attitudes and behaviors (Walumbwa and Hartnell, 2011). In support of this theoretical view, empirical studies have shown that leader identification, or the degree to which employees define themselves in relation to their leader, mediates the effects of leadership on employee attitudes and behaviors (Zhu et al., 2013). Employees who identify strongly with their leader may internalize their values and align with their goals, thereby reducing the likelihood of deviant behaviors. However, if identification is low, especially under perceived coercion or unfairness, the risk of counterproductive work behavior may increase.

Another important factor influencing leader identification is traditionality, which reflects an individual’s endorsement of hierarchical authority (Li et al., 2018). Traditionality refers to organized cognitive attitudes, ideas, value orientations, temperament characteristics, and behavioral wills of individuals in traditional societies (Spreitzer et al., 2005). It is viewed as the most accurate representation of traditional Chinese characteristics and value perspectives (Hui et al., 2004b), which encompasses the individual’s acknowledgment of traditional Confucian principles, including deference to authority, adherence to ethical conduct, self-preservation, respect for one’s parents, reverence for ancestors, and male dominance (Xiong Chen and Aryee, 2007). For traditionalists, leaders are seen as paternal figures and followers as their children. The expectation is that followers should trust and demonstrate loyalty to their leaders (Rarick, 2007). Therefore, traditionality tends to influence leaders and followers, like father-son relationships (Hui et al., 2004a). It is noteworthy that the adverse implications of authoritarianism can be either mitigated or reshaped by moderating factors, with the traditionality of employees serving as one such moderator (Farh et al., 2014).

### Hypothesis development

#### Paternalistic leadership and counterproductive work behavior

Leadership styles substantially impact counterproductive work behavior, and the prevailing conflict between Gen Z employees and organizations frequently centers on their preferences for leadership styles (Ogunsola et al., 2024). Prior research has highlighted the beneficial impact of leaders’ benevolence and morality on team identification and top management team decision efficacy, whereas authoritarianism is found to have deleterious consequences in these contexts (Chen et al., 2015). Similarly, Zhang S. et al. (2021) found that benevolent and moral leadership positively influenced safety participation among Chinese high-speed railway drivers. In contrast, authoritarian leadership styles have been associated with undesirable effects in these domains. Given the personality traits of Gen Z employees, they typically resist commanding and authoritarian leadership styles, instead favoring leadership characterized by benevolence and moral integrity (Nikolic, 2022).

Authoritarian leadership, characterized by rigidity and control, tends to undermine positive employee behaviors and elicit adverse psychological and behavioral reactions. For example, Liu and Ling (2025) found that authoritarian leadership increased emotional exhaustion and reduced voice behavior (when employees speak up with ideas or concern to improve work or prevent problems) among frontline service workers in China. Likewise, Zheng et al. (2025) reported that authoritarian leadership significantly undermined young nurses’ psychological capital and increased burnout. Authoritarian leadership tends to increase counterproductive work behavior among Gen Z employees, as its rigid, control-oriented style conflicts with their preference for autonomy and fairness (Luqman et al., 2020). Taken together, these findings underscore the relevance of authoritarian leadership as a potential predictor of counterproductive work behavior among Gen Z employees. In our study, we propose that authoritarian leadership will be positively associated with counterproductive work behavior among Gen Z employees.

On the contrary, leaders’ benevolence and morality exhibit correlations with favorable outcomes, including trust in managers, manifestations of organizational citizenship behavior, and the fostering of creativity (Chen et al., 2014). Benevolent leadership reduces counterproductive work behavior among Gen Z employees by demonstrating personalized care and support, which fosters emotional bonds and a sense of being valued, thereby discouraging retaliatory or disengaged behaviors (Luqman et al., 2020). Benevolent leadership has also been shown to increase work engagement and initiative, thereby decreasing counterproductive behavior (Li et al., 2022). Moral leadership, through its emphasis on fairness, integrity, and ethical role modeling, enhances perceptions of justice and trust (Mohi Ud Din and Zhang, 2023), which in turn decreases the likelihood of norm-violating actions and promotes constructive employee conduct. Also, this leadership has been found to negatively correlate with various forms of counterproductive work behavior, including abuse, withdrawal, theft, sabotage, and production/service deviance (Kulualp and Koçoğlu, 2019). Together, these two leadership dimensions serve as protective factors that buffer Gen Z employees against engaging in counterproductive work behavior. In our study, we propose that both benevolent and moral leadership will be negatively associated with counterproductive work behavior among Gen Z employees.

Based on the literature reviewed above, the following main hypotheses are developed.

*H1a*: Authoritarian leadership is positively related to Chinese Gen Z employees’ counterproductive work behavior.

*H1b:* Benevolent leadership is negatively related to Chinese Gen Z employees’ counterproductive work behavior.

*H1c:* Moral leadership is negatively related to Chinese Gen Z employees’ counterproductive work behavior.

#### The mediating role of leader identification

Gen Z employees generally prefer a leadership approach that involves seeking consensus rather than giving commands. They prefer encouragement and participation over being autocratic, and value adaptability and flexibility over rigidity and hierarchy (McCrindle and Fell, 2019). Therefore, authoritarian leadership focuses on the leaders’ dominance, rigid oversight, and the subordinates’ unquestioning adherence (Zhang and Xie, 2017) is likely to negatively evaluate authoritarian leaders. Integrating the social identity theory and characteristics of both Gen Z employees and leadership styles, we argue that authoritarian leadership will result in lower leadership identification. Gen Z employees, who value autonomy, inclusivity, and open communication, may find authoritarian leadership, which is marked by strict control and top-down decision-making, misaligned with their values (Demirbilek and Keser, 2022). This misalignment can hinder positive identification with their leaders, resulting in a weaker sense of belonging and loyalty. Consequently, authoritarian leadership is likely to lead to lower leadership identification among Gen Z employees. By contrast, compared with authoritarian leadership, benevolent and moral leadership typically emphasize care, support, and a focus on morals, which aligns with the values that Gen Z employees themselves often appreciate (Nikolic, 2022). Gen Z values transparency, ethical behavior, and a supportive work environment, which makes them more likely to resonate with leaders who exhibit these qualities. They appreciate leaders who are honest and open in their communication, act with integrity, and create a positive workplace. This approach is especially inclusive of Gen Z employees, making them more likely to embrace and identify with this style of leadership. Based on this, we make the following assumptions:

*H2a:* Authoritarian leadership is negatively related to Chinese Gen Z employees’ leader identification.

*H2b*: Benevolent leadership is positively related to Chinese Gen Z employees’ leader identification.

*H2c*: Moral leadership is positively related to Chinese Gen Z employees’ leader identification.

Employees who strongly identify with their leaders tend to exhibit greater attentiveness and loyalty toward both their supervisors and organizations (Sluss et al., 2012). Such employees are more likely to adopt their leaders’ priorities, objectives, and values, often reshaping their own self-concept to align with their leaders’ standards, beliefs, and behaviors (Gu et al., 2015). This strong identification fosters a sense of obligation to contribute constructively, including the willingness to share innovative ideas and solutions with their leaders (Liu et al., 2010).

Moreover, employees who identify closely with their leaders are typically more motivated and driven to meet their leaders’ expectations. They often engage in behaviors that not only benefit the leader but also support organizational goals (Johnson, 2010). When leaders are perceived as role models or relatable figures, their actions and attitudes have a stronger influence on employees’ behaviors (Wang and Rode, 2010).

Drawing on this perspective, we propose that employees with a high level of leader identification are less likely to engage in counterproductive work behaviors, regardless of how they are treated by their leaders. In this context, leader identification acts as a protective factor, mitigating negative workplace behaviors. Taken together, previous research suggests that leader identification plays a critical mediating role in this relationship, exerting a negative influence on employees’ counterproductive work behavior. Therefore, we propose the following hypotheses:

*H3:* Leader identification is negatively related to Chinese Gen Z employees’ counterproductive work behavior.

*H4a*: Leader identification mediates the relationship between authoritarian leadership and Chinese Gen Z employees’ counterproductive work behavior.

*H4b:* Leader identification mediates the relationship between benevolent leadership and Chinese Gen Z employees’ counterproductive work behavior.

*H4c:* Leader identification mediates the relationship between moral leadership and Chinese Gen Z employees’ counterproductive work behavior.

#### The moderating role of traditionality

We argue that traditionality moderates the relationship between paternalistic leadership and leadership identification. Individuals with high traditionality have more recognition of ethics and leadership authority, while individuals with low traditionality tend to pursue effectiveness and equality (Farh et al., 2007). High-traditional individuals pay attention to traditional cultural values such as benevolence, righteousness, morality, and self-discipline (Li et al., 2017), which can have a positive impact on their sense of leader identification and improve the influence of paternalistic leadership. On the contrary, individuals with low adherence to traditional values are less likely to embrace or even pay attention to the ethical norms promoted by traditional culture (Wu et al., 2021). They also tend to question or disregard the authority and status of leaders (Liu et al., 2013) As a result, the influence of paternalistic leadership may be weakened among these individuals.

Based on Gen Z employees’ traits, we assume those with higher levels of traditionality tend to exhibit a greater degree of identification with any type of leadership. This implies that regardless of the actions taken by leaders, Gen Z employees are inclined to accept them and feel a sense of identification with the hierarchical relationship (Hui et al., 2004a). In contrast, Gen Z employees with lower levels of traditionality tend to have higher expectations of leadership styles. For these individuals, they prefer to collaborate with leaders who encourage participation and adaptability, rather than those who enforce strict hierarchical structures. They are more likely to identify with this open and egalitarian work environment, leading to higher levels of job satisfaction and positivity (Bălan and Vreja, 2018). Hence, we argue that traditionality moderates the relationship between paternalistic leadership and leader identification, therefore, the theoretical assumption is as follow:

*H5a*: The negative relationship between authoritarian leadership and leader identification is moderated by traditionality, such that it is stronger for lower than for higher levels of traditionality.

*H5b*: The positive relationship between benevolent leadership and leader identification is moderated by traditionality, such that it is stronger for lower than for higher levels of traditionality.

*H5c*: The positive relationship between moral leadership and leader identification is moderated by traditionality, such that it is stronger for lower than for higher levels of traditionality.

**Figure 1:**
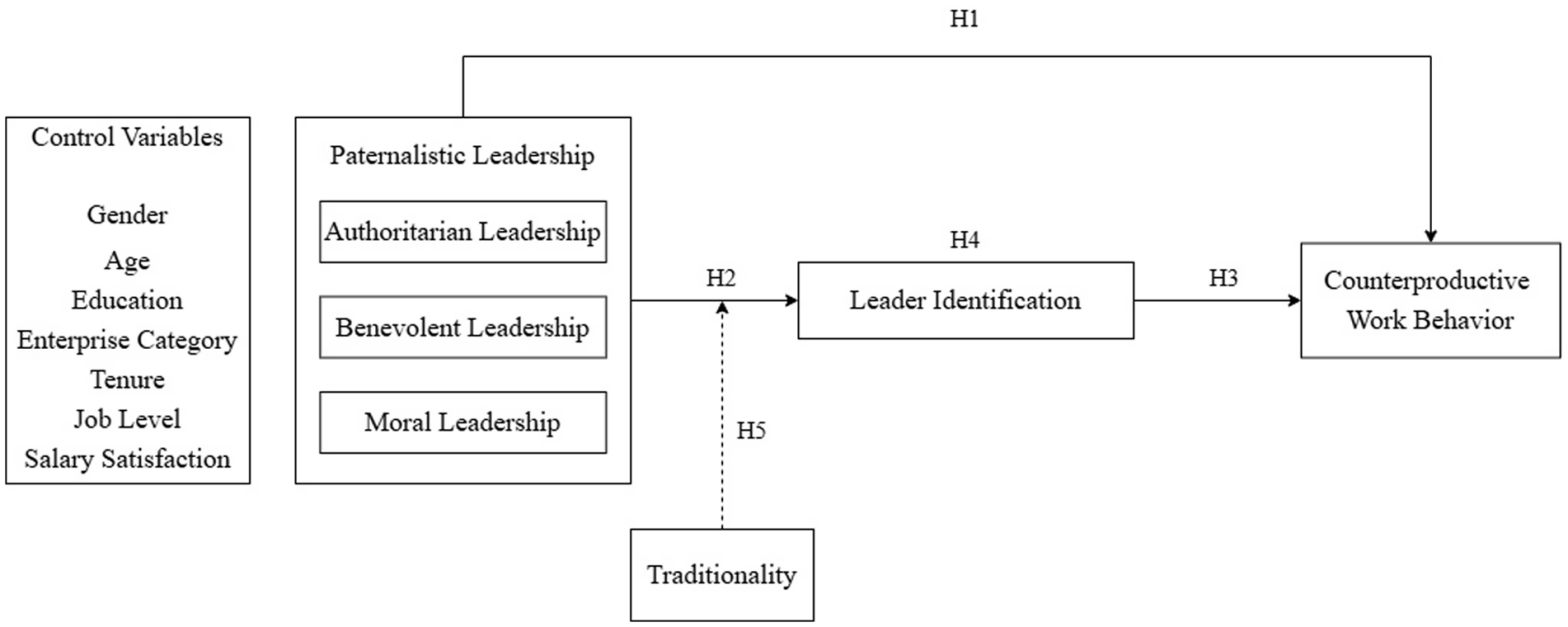
Theoretical model.

Based on the above discussion, the moderated mediation model of the relationship between paternalistic leadership and the Gen Z employees’ counterproductive work behavior was constructed, as shown in Figure 1.

## Methods

### Participants and procedures

We utilized an online survey to collect data and test our hypotheses, focusing on Gen Z employees in Mainland China. To minimize the potential impact of common method variance, we collected the data at two separate points in time, aligning with the proposed theoretical model (Podsakoff et al., 2012). At Time one (T1), Gen Z employees reported perceived leadership styles, including paternalistic leadership and control variables (i.e., authoritarian leadership, benevolent leadership, and moral leadership). They were also asked to rate their own degree identification with leaders at the same time one (T1). At Time two (T2), 1 months later, the T1 respondents were asked to rate their level of the counterproductive work behavior and traditionality again.

The survey was administered through the Credamo platform (a questionnaire survey platform based in mainland China). We employed a convenience sampling approach. Questionnaire links were distributed with the help of colleagues, senior students working in various companies, and advisors. Before participation, respondents were informed of the research purpose, assured that their data would remain confidential and used solely for academic research, and encouraged to reach out via telephone, WeChat, or SMS if they had any questions during completion.

By the final submission deadline, a total of 358 responses were recorded through the online questionnaire platform. During data processing, we excluded responses with completion times of less than 60 s, those exhibiting uniform answers across all items, and any other invalid data submissions. Ultimately, 324 valid responses were collected. In this study, statistical analysis of the data was primarily conducted using Stata 17.0. Initially, Stata 17.0 was employed to perform descriptive analyses on both demographic statistics and the four key research variables, providing an overview of the data as a whole.

Table 1 presents the key demographic statistics of the participants. It shows males represent 53.09% and females 46.91% of respondents. Most Gen Z employees (53.40%) are aged 27–29. In terms of education, 48.15% hold bachelor’s degrees, 16.36% have graduate degrees, and 15.74% have high school education or lower. Private enterprises employ 28.09% of respondents, and the majority (48.15%) have 1–2 years of work experience. Regular employees make up 61.42% of respondents, reflecting their limited experience and younger age. Additionally, 73.6% express satisfaction with their current salary levels.

**Table 1:** Descriptive statistics of demographic information.

Demographic information		N	%
Gender	Male	172	53.09%
	Female	152	46.91%
Age	21–23	13	4.01%
	24–26	101	31.17%
	27–29	173	53.40%
	30–32	37	11.42%
Education	High School and Below	51	15.74%
	Higher Professional School	64	19.75%
	Bachelor Degree	156	48.15%
	Master Degree / Ph. D.	53	16.36%
Enterprise Category	Public Institution	45	13.89%
	State-Owned Enterprise	63	19.44%
	Private Enterprise	91	28.09%
	Sino-Foreign Joint Venture	55	16.98%
	Foreign-Invested Enterprise	40	12.35%
	Others	30	9.26%
Tenure	0–12 Months	72	22.02%
	13–24 Months	156	48.15%
	25–36 Months	68	20.99%
	37 Months and Above	28	8.64%
Job Level	General Staff	199	61.42%
	Frontline Managers	53	16.36%
	Middle Managers	36	11.11%
	Senior Managers	36	11.11%
Salary Satisfaction	Very Dissatisfied	35	10.80%
	Dissatisfied	51	15.74%
	Neutral	80	24.69%
	Satisfied	82	25.31%
	Very Satisfied	76	23.46%

### Measures

All measurement scales involved in our study were adapted from the existing literature and were employed and demonstrated to have good reliability and validity by many previous studies in the Chinese context (e.g., Farh et al., 2008; Yu and Zhang, 2007; Liu et al., 2013; Chen and Spector, 2011). All multi-item measures were rated on a five-point Likert-type scale ranging from 1 (strongly disagree) to 5 (strongly agree).

*Paternalistic leadership (T1)*. At Time 1, for paternalistic leadership, we used a 15-items scale developed by Cheng to measure paternalistic leadership (Cheng et al., 2003). The sample items for authoritarianism, benevolence, and morality included: “My supervisor asks me to obey his/her instructions completely,” and “My supervisor is a virtuous leader compared to other company leaders.”

*Leader identification (T1).* At Time 1, we used Walumbwa and Hartnell’s 10-items measure to assess how employees identify with their direct leader (Walumbwa and Hartnell, 2011). This scale was originally designed to measure employees’ identification with the organization (Kark et al., 2003); in this study, items focused on the direct leader of the employee. Two example items are, “When someone criticizes my direct leader, it feels like an insult to me” and “I am proud to tell others I work with this supervisor.”

*Traditionality (T2).* At Time 2, participants rated their traditionality using the 5-item scale by Farh et al. (1997). Two sample items are “The best way to avoid mistakes is to follow the instructions of a senior person” and “When people are in dispute, they should ask the most senior person to decide who is right.”

*Counterproductive work behavior (T2).* At Time 2, members rate their own degree of counterproductive work behavior. The counterproductive work behavior was assessed using a 10-item Chinese version derived from Bennett and Robinson (2000) original 19-item scale. This shorter version has been validated in Chinese organizational research and has been widely adopted in local studies. In this study, two sample items are “Neglected to follow your boss’s instructions” and “Put little effort into your work.”

## Results

### Reliability and validity test results

To assess the potential concern of common method bias (CMB), we conducted Harman’s single-factor test by loading all study items into an unrotated principal component analysis. The results showed that the first factor accounted for 47.65% of the total variance, which exceeds the commonly used 40% threshold but falls below the more conservative 50% threshold, suggesting that CMB may not be a significant concern (Podsakoff et al., 2003; Kock et al., 2021). Moreover, following recommendations in the literature, we adopted several procedural remedies to further minimize CMB, including assuring participant anonymity, randomizing the presentation order of items, and using varied scale formats to reduce evaluation apprehension and response consistency effects (Podsakoff et al., 2003; Kock et al., 2021).

As shown in Table 2, the three dimensions of paternalistic leadership (authoritarian, benevolent, and moral leadership) demonstrated strong internal consistency, with Cronbach’s alpha values of 0.937, 0.928, and 0.931, respectively. All values exceed the recommended threshold of 0.80, indicating high reliability of the measurement scales (Nunnally and Bernstein, 1994). Similarly, the Cronbach’s alpha for leader identification, traditionality, and the counterproductive work behavior are 0.955, 0.917, and 0.921, thereby inferring robust internal consistency and high reliability across the variable indicators. Analysis from Table 2 also reveals that the Average Variance Extracted (AVE) for authoritarian leadership, benevolent leadership, moral leadership, leader identification, and traditionality all exceed 0.7, while AVE for the counterproductive work behavior slightly surpasses 0.5, meeting the required standard (Fornell and Larcker, 1981). The Composite Reliability (CR) scores all exceed 0.9, indicating a strong performance (Bagozzi and Yi, 1988). Factor loading values range from 0.722 to 0.912 (surpassing 0.6), providing further evidence of the scale’s sound convergent validity (Hair et al., 2010).

**Table 2:** Reliability and validity statistics of variables.

Variable	AVE	CR	KMO	Cronbach’s α
AL	0.799	0.952	0.896	0.937
BL	0.777	0.946	0.888	0.928
ML	0.784	0.948	0.906	0.931
LI	0.715	0.962	0.967	0.955
T	0.752	0.938	0.895	0.917
CWB	0.585	0.934	0.941	0.921

AL, authoritarian leadership; BL, benevolent leadership; ML, moral leadership; LI, leader identification; T, traditionality; CWB, counterproductive work behavior.

Confirmatory factor analysis was conducted to assess discriminant validity among paternalistic leadership, leader identification, traditionality, and the counterproductive work behavior. As shown in Table 3, the results for the six-factor model yielded χ^2^ = 1001.319, *df* = 725, χ^2^/*df* = 1.381 < 3, RMSEA = 0.034 < 0.08, TLI = 0.973 > 0.9, CFI = 0.975 > 0.9, and SRMR = 0.034 < 0.08. They indicate a good overall model fit for the six-factor model, and its fit indices outperform those of the other factor models, demonstrating the satisfactory discriminant validity of the scale used in this study.

**Table 3:** Results of confirmatory factor analysis.

Model	χ^2^	*df*	χ^2^/*df*	RMSEA	TLI	CFI	SRMR
Six-Factor Model	1001.319	725	1.381	0.034	0.973	0.975	0.034
Five-Factor Model	1630.452	730	2.233	0.062	0.912	0.917	0.049
Four-Factor Model	2254.756	734	3.072	0.080	0.852	0.860	0.069
Three-Factor Model	2664.172	737	3.615	0.090	0.813	0.823	0.081
Two-Factor Model	4034.903	739	5.460	0.118	0.681	0.698	0.106
One-Factor Model	4747.962	741	6.408	0.129	0.613	0.632	0.153

*N* = 324; Six-Factor Model: AL, BL, ML, LI, T, CWB; Five-Factor Model: AL, BL+ML, LI, T, CWB; Four-Factor Model: AL+BL+ML, LI, T, CWB; Three-Factor Model: AL+BL+ML, LI+T, CWB; Two-Factor Model: AL+BL+ML+LI+T, CWB; One-Factor Model: AL+BL+ML+LI+T + CWB. χ^2^/df < 3; RMSEA < 0.08; TLI > 0.9; CFI > 0.9; SRMR < 0.08.

### Correlation analysis results

Table 4 summarizes the mean values, standard deviations, and correlation coefficients. The analysis reveals the negative relationship between benevolent leadership and the counterproductive work behavior (*r* = −0.473, *p* < 0.01). Moral leadership is negatively related to the counterproductive work behavior (*r* = −0.589, *p* < 0.01). And there was a positive relationship between authoritarian leadership and the counterproductive work behavior (*r* = 0.656, *p* < 0.01).

**Table 4:** Descriptive statistics and correlations.

Variable name	Mean	SD	1	2	3	4	5	6	7	8	9	10	11	12
1. Gender	1.469	0.500												
2. Age	27.114	1.931	−0.011											
3. Education	2.651	0.934	−0.146***	−0.071										
4. Enterprise Category	3.222	1.485	0.097*	0.045	−0.216***									
5. Tenure	22.503	11.798	0.033	0.110**	−0.088	0.136**								
6. Job Level	1.719	1.046	0.075	0.260***	−0.218***	0.250***	−0.113**							
7. Salary Satisfaction	3.349	1.290	−0.029	−0.180***	−0.027	−0.050	0.080	−0.124**						
8. AL	3.336	1.184	−0.011	−0.053	0.166***	−0.063	0.036	−0.156***	0.205***					
9. BL	2.790	1.105	0.040	−0.013	−0.041	0.012	−0.049	0.019	0.031	−0.691***				
10. ML	2.988	1.158	−0.012	−0.055	−0.099*	0.084	0.070	0.025	0.071	−0.601***	0.637***			
11. LI	3.019	1.034	0.025	−0.030	−0.084	0.136**	0.010	−0.006	0.198***	−0.486***	0.568***	0.712***		
12. Traditionality	2.865	1.048	0.026	0.000	−0.012	0.089	0.027	−0.066	0.293***	−0.230***	0.392***	0.435***	0.753***	
13. CWB	2.773	0.837	0.033	−0.022	0.121**	−0.117**	0.014	−0.064	0.096*	0.656***	−0.473***	−0.589***	−0.746***	−0.469***

*N* = 324. *p < 0.05. **p < 0.01. ***p < 0.001. Gender was coded as 1 = male, 2 = female. Age: 1 = “21–23”, 2 = “24–26”, 3 = “27–29”, 4 = “30–32.” Education: 1 = “High School and Below”, 2 = “Higher Professional School”, 3 = “Bachelor Degree”, 4 = “Master Degree / Ph. D.” Enterprise: 1 = “Public Institution”, 2 = “State-Owned Enterprise”, 3 = “Private Enterprise,” 4 = “Sino-Foreign Joint Venture,” 5 = “Foreign-Invested Enterprise,” 6 = “Others.” Tenure: 1 = “0–12 Months,” 2 = “13–24 Months,” 3 = “25–36 Months,” 4 = “37 Months and Above.” Job Level: 1 = “General Staff,” 2 = “Frontline Managers,” 3 = “Middle Managers,” 4 = “Senior Managers.” Salary Satisfaction is measured on the Likert five-point scale, ranging from “very dissatisfied” to “very satisfied”.

Both benevolent leadership (*r* = 0.568, *p* < 0.01) and moral leadership (*r* = 0.712, *p* < 0.01) are positive related to leader identification. In contrast, the table indicates a negative relationship between authoritarian leadership and leader identification. (*r* = −0.486, *p* < 0.01). Finally, there was a negative interaction between leader identification and the counterproductive work behavior (*r* = −0.746, *p* < 0.01). This preliminary analysis suggests a significant negative relationship between leader identification and employees’ counterproductive work behavior.

### Multiple regression analysis

Table 5 presents regression models of paternalistic leadership and its dimensions on employees’ counterproductive work behavior and leader identification. In Model 2, authoritarian leadership shows a positive impact on employees’ counterproductive work behavior (*b* = 0.470, *p* < 0.001), supporting Hypothesis *H1a*. Model 3 reveals that benevolent leadership is negatively related to employees’ counterproductive work behavior (*b* = −0.359, *p* < 0.001), supporting Hypothesis *H1b*. In Model 4, moral leadership significantly influences employees’ counterproductive work behavior, demonstrating a negative interaction (*b* = −0.428, *p* < 0.001), supporting Hypothesis *H1c*. In Model 7, authoritarian leadership shows a negative impact on leader identification (*b* = −0.489, *p* < 0.001), confirming Hypothesis *H2a*. Model 8 indicates a positive relationship between benevolent leadership and leader identification (*b* = 0.524, *p* < 0.001), supporting Hypothesis *H2b*. In Model 9, moral leadership positively affects leader identification (*b* = 0.627, *p* < 0.001), confirming Hypothesis *H2c.* Finally, according to Model 5, it is evident that leader identification exerts a negative impact on employees’ counterproductive work behavior (*b* = −0.643, *p* < 0.001), thereby supporting Hypothesis *H3*.

**Table 5:** Multiple regression results predicting counterproductive work behavior and leader identification.

Variable	CWB					LI			
	Model 1	Model 2	Model 3	Model 4	Model 5	Model 6	Model 7	Model 8	Model 9
Gender	0.102	0.075	0.131	0.070	0.119*	0.025	0.054	−0.016	0.074
Age	0.003	−0.002	0.002	−0.014	0.006	0.005	0.011	0.007	0.031
Education	0.102*	0.008	0.086	0.057	0.062	−0.061	0.036	−0.038	0.004
Enterprise Category	−0.054*	−0.054*	−0.052	−0.034	0.012	0.102**	0.103**	0.099**	0.073*
Tenure	0.002	0.001	−0.000	0.004	−0.000	−0.003	−0.002	−0.001	−0.007*
Job Level	−0.005	0.049	−0.003	0.007	−0.029	−0.037	−0.094	−0.040	−0.055
Salary Satisfaction	0.061*	−0.025	0.072*	0.083**	0.166***	0.163***	0.253***	0.147***	0.131***
AL		0.470***					−0.489***		
BL			−0.359***					0.524***	
ML				−0.428***					0.627***
LI					−0.643***				
*R^2^*	0.037	0.443	0.260	0.378	0.627	0.065	0.352	0.377	0.546
*ΔR^2^*	0.015	0.429	0.241	0.362	0.618	0.044	0.336	0.361	0.534
*F*	1.72	31.36***	13.86***	23.95***	66.31***	3.13**	21.43***	23.82***	47.33***

*N* = 324. *p < 0.05. **p < 0.01. ***p < 0.001. AL, authoritarian leadership; BL, benevolent leadership; ML, moral leadership; LI, leader identification; CWB, counterproductive work behavior.

### Mediation and moderation analysis

Table 6 examines the mediating effect of identification in the relationship between authoritarian leadership, benevolent leadership, and moral leadership and counterproductive work behavior. Bootstrapping with 5,000 resamples was employed to enhance the robustness of the estimates. This method was chosen because it provides robust estimates of indirect effects, even when the normality assumption is violated, which is common in complex mediation models. Additionally, bootstrap resampling enhances the accuracy of confidence intervals, making it particularly suitable for capturing the nuances of leader identification’s mediating role (Hayes, 2017).

**Table 6:** Testing for mediation effects of leader identification.

Path	Effect	Estimate	BootSE	BootLLCI	BootULCI	Mediation
AL → LI → CWB	Indirect Effect	0.245***	0.028	0.189	0.300	Partial Mediation
	Direct Effect	0.225***	0.033	0.160	0.290	
BL → LI → CWB	Indirect Effect	−0.326***	0.029	−0.384	−0.268	Full Mediation
	Direct Effect	−0.032	0.032	−0.096	0.030	
ML → LI → CWB	Indirect Effect	−0.378***	0.028	−0.433	−0.323	Full Mediation
	Direct Effect	−0.050	0.035	−0.118	0.018	

*N* = 324. *p < 0.05. **p < 0.01. ***p < 0.001. Resampling times = 5,000. AL, authoritarian leadership; BL, benevolent leadership; ML, moral leadership; LI, leader identification; CWB, counterproductive work behavior.

Authoritarian leadership facilitates employees’ counterproductive work behavior through leader identification, as indicated by a significant indirect effect (95% CI [0.189, 0.300], excluding zero), while the direct effect is not significant (95% CI [0.160, 0.290], including zero). This implies that leader identification partially mediates the influence of authoritarian leadership on employees’ counterproductive work behavior. Thereby, it can confirm Hypothesis *H4a*. Benevolent leadership reduces employees’ counterproductive work behavior through leader identification, as indicated by a significant indirect effect (95% CI [−0.384, −0.027], excluding zero), while the direct effect is not significant (95% CI [−0.096, 0.030], including zero). This indicates that leader identification fully mediates the impact of benevolent leadership on employees’ counterproductive work behavior, supporting Hypothesis *H4b*. Last, moral leadership reduces employees’ counterproductive work behavior through leader identification, as indicated by a significant indirect effect (95% CI [−0.433, −0.323], excluding zero), while the direct effect is not significant (95% CI [−0.118, 0.018], including zero). Thus, leader identification fully mediates the influence of moral leadership on employees’ counterproductive work behavior, supporting Hypothesis *H4c*.

In order to examine the moderating effect of traditionality on the relationship between the three dimensions of paternalistic leadership and leader identification, the interaction of authoritarian leadership with traditionality, benevolent leadership with traditionality, and moral leadership with traditionality were sequentially incorporated into the model. Regression analyses were conducted on leader identification, and the results are presented in the table.

**Figure 2:**
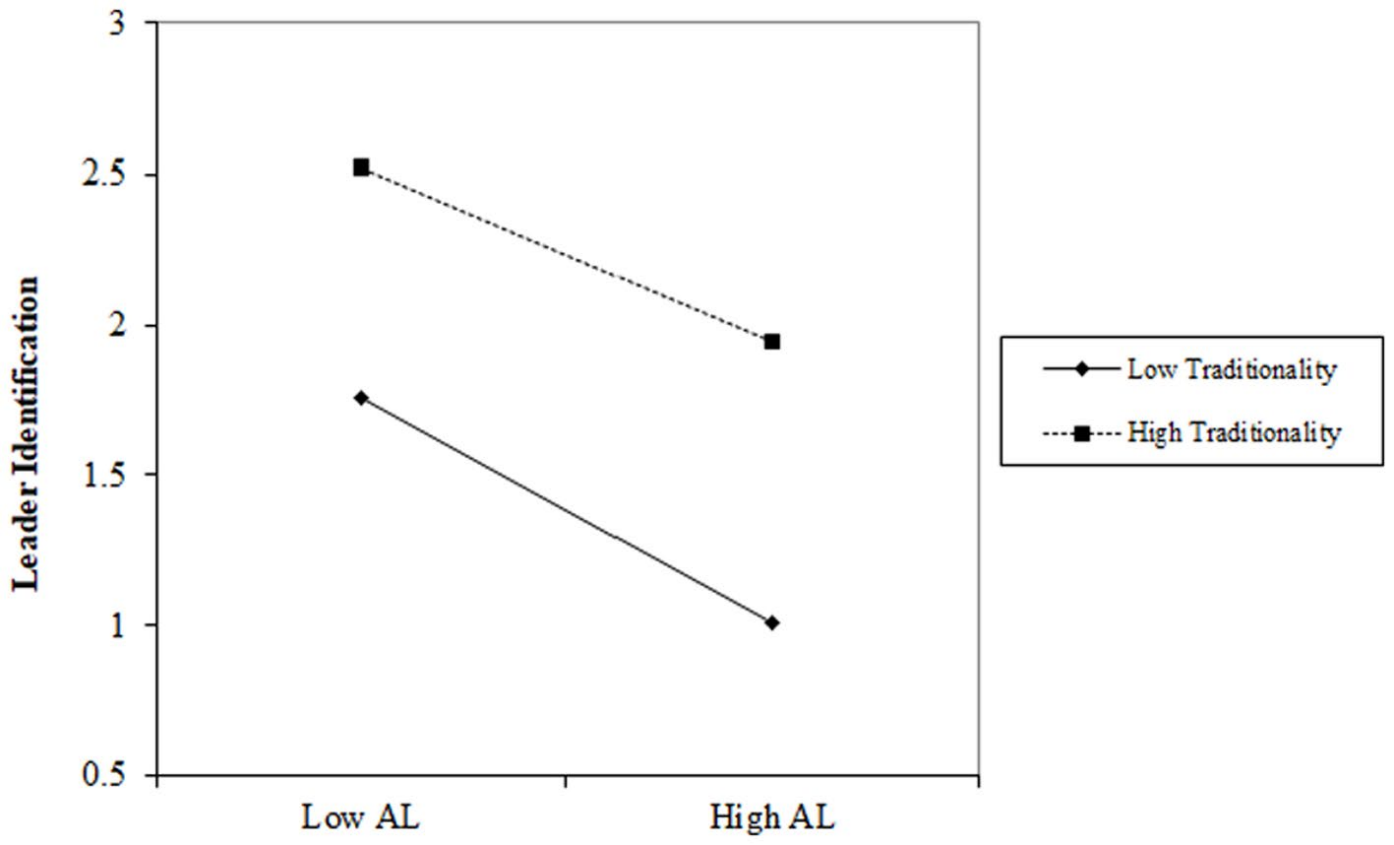
Moderation effect of traditionality on the relationship between authoritarian leadership and leader identification.

As summarized by Model 1 in Table 7, the influence of authoritarian leadership on leader identification is negative (*b* = −0.317, *p* < 0.001). The interaction between authoritarian leadership and traditionality impacts employees’ counterproductive work behavior positively (*b* = 0.071, *p* < 0.05). This suggests that when traditionality is high, the negative relationship between authoritarian leadership and leader identification tends to be weaker. Conversely, when traditionality is low, this negative relationship is stronger. To better visualize the moderation effect, we plotted the interaction terms in Figure 2.

**Table 7:** Moderation effects of traditionality on the relationship between paternalistic leadership and leader identification.

Variable	LI		
	Model 1	Model 2	Model 3
Gender	0.009	−0.042	0.020
Age	−0.014	−0.015	0.006
Education	−0.004	−0.048	−0.015
Enterprise Category	0.041	0.038	0.035
Tenure	−0.001	0.000	−0.004
Job Level	−0.027	0.014	−0.011
Salary Satisfaction	0.068*	0.000	0.010
AL	−0.317***		
BL		0.327***	
ML			0.436***
T	0.611***	0.587***	0.511***
AL*T	0.071*		
BL*T		−0.117***	
ML*T			−0.083**
*R^2^*	0.689	0.681	0.764
*ΔR^2^*	0.679	0.670	0.756
*F*	69.21***	66.69***	101.04***

*N* = 324. *p < 0.05. **p < 0.01. ***p < 0.001. AL, authoritarian leadership; BL, benevolent leadership; ML, moral leadership; LI, leader identification; T, traditionality; CWB, counterproductive work behavior.

In Model 2, the influence of benevolent leadership on leader identification is positive (*b* = 0.327, *p* < 0.001). The interaction between benevolent leadership and traditionality significantly impacts employees’ counterproductive work behavior negatively (*b* = −0.117, *p* < 0.001). In Model 3, the influence of moral leadership on leader identification is positive (*b* = 0.436, *p* < 0.001). The interaction between moral leadership and traditionality significantly impacts employees’ counterproductive work behavior negatively (*b* = −0.083, *p* < 0.01). This implies that varying levels of traditionality affect the relationship between benevolent leadership, moral leadership and leader identification. When traditionality is high, the positive relationship between benevolent leadership and moral leadership and leader identification tends to diminish. Conversely, when traditionality is low, the negative relationship between benevolent leadership and moral leadership and leader identification intensifies.

**Figure 3:**
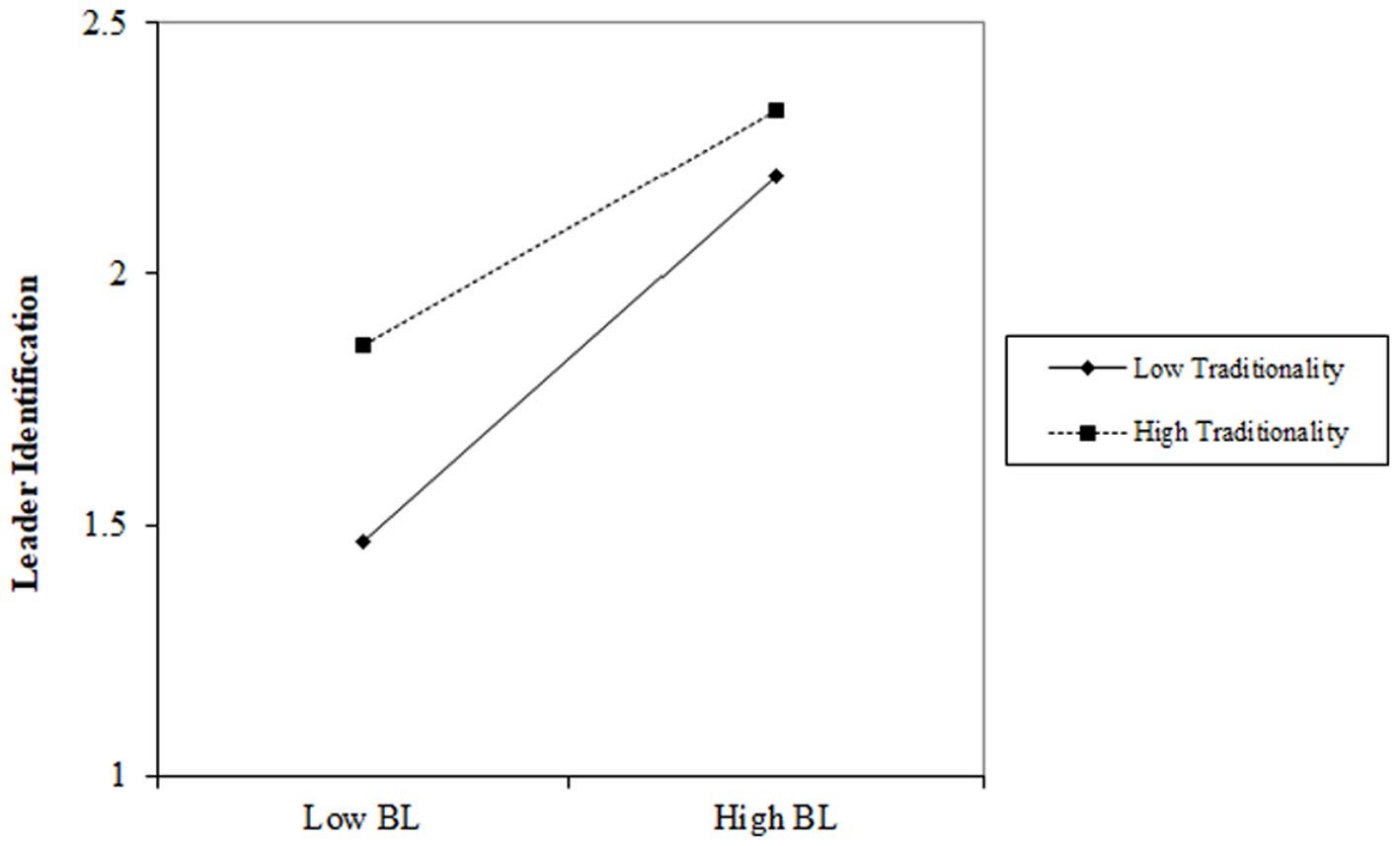
Moderation effect of traditionality on the relationship between benevolent leadership and leader identification.

**Figure 4:**
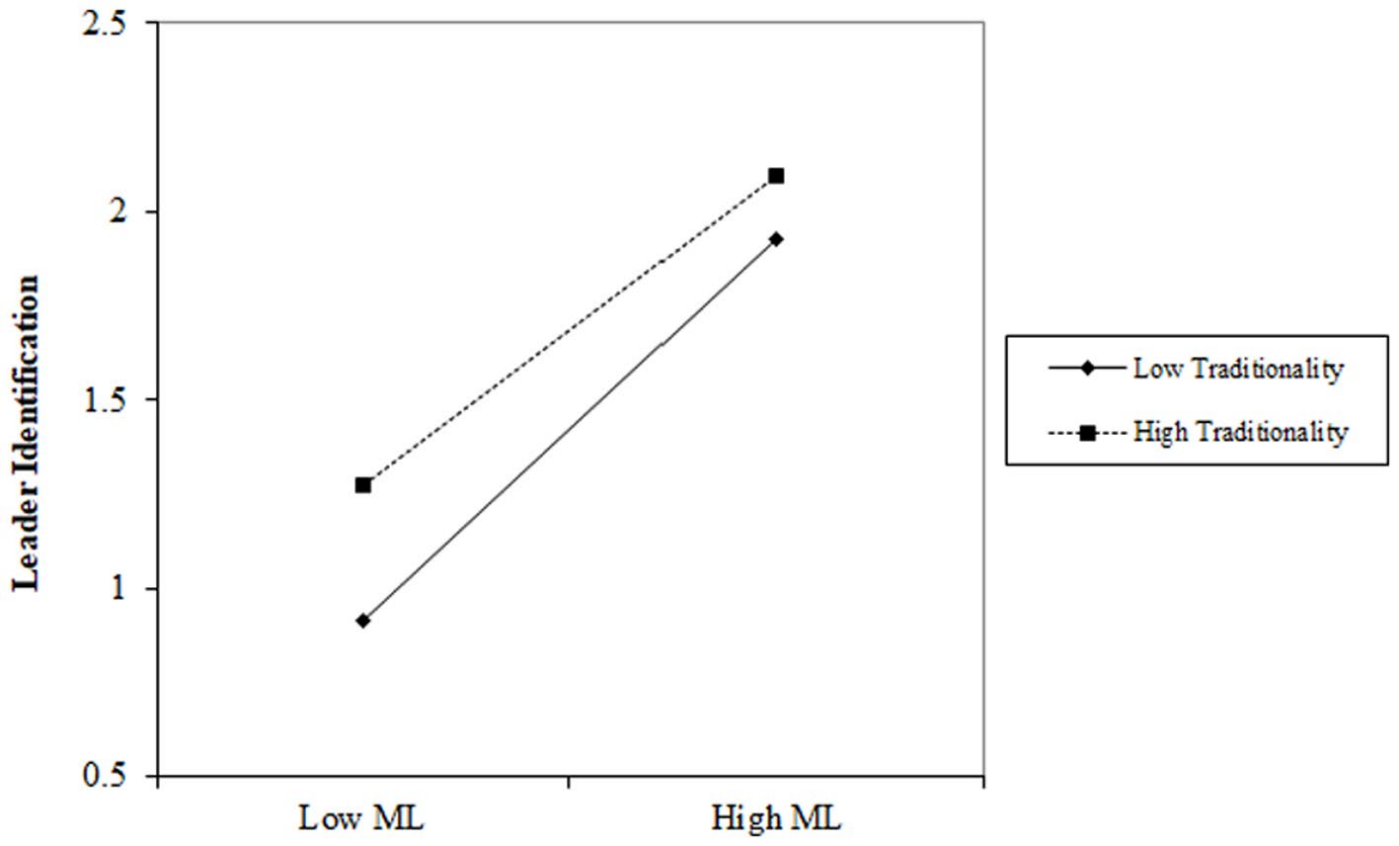
Moderation effect of traditionality on the relationship between moral leadership and leader identification.

As depicted in Figures 3, 4, in the relationship between benevolent leadership, moral leadership, and leader identification, the slope of low-level traditionality is steeper compared to high-level traditionality. Consequently, lower traditionality reinforces the positive correlation between benevolent leadership, moral leadership, and leader identification. With a decrease in traditionality, the positive impact of benevolent leadership and moral leadership on leader identification also strengthens.

## Discussion

### Theoretical implications

This study develops a theoretical model that explores how paternalistic leadership affects Chinese Gen Z employees’ counterproductive work behavior, with leader identification as a mediator and traditionality as a moderator. Our findings support the three main hypotheses: the impact of paternalistic leadership on counterproductive work behavior, the mediating role of leader identification, and the moderating role of traditionality.

First, this study offers important insights into how distinct dimensions of paternalistic leadership shape workplace behavior among Chinese Gen Z employees. Regarding each dimension, benevolent leadership and moral leadership exhibit significant negative effects on employee’s counterproductive work behavior, whereas authoritarian leadership shows a significant positive effect. Benevolent leadership demonstrates sincere treatment toward individuals, offering substantial assistance and care, which makes employees feel valued by the leadership, consequently reducing the likelihood of generating more counterproductive work behavior. Moral leadership influences employees through its moral appeal, fostering a greater willingness among employees to personally invest in their work, thereby reducing substantial counterproductive work behavior. However, authoritarian leadership’s demeanor tends to hurt employees emotionally, dampening their enthusiasm and motivation for work, thereby stimulating younger employees to engage in more counterproductive work behavior.

Secondly, the results show that leader identification fully mediates the relationship between benevolent leadership and moral leadership and employees’ counterproductive work behavior, and partially mediates the relationship between authoritarian leadership and the counterproductive work behavior. This indicates that Gen Z employees are more inclined to endorse the positive leadership styles of benevolent leadership and moral leadership, which foster positive self-value feedback among employees, consequently reducing their negative attitudes toward work and content innovation. Conversely, Gen Z employees are more resistant to the influence of authoritarian leadership, displaying lower identification, thereby prompting more counterproductive work behavior. This is mainly because authoritarian leadership typically emphasizes power and control, which contradicts the values cherished by Gen Z employees, such as respecting individual rights and the diversity of values (Dolot, 2018). The conflict resulting from this clash of values leads to lower identification, thereby increasing the occurrence of the counterproductive work behavior. The emphasis on care, support, and moral principles typically highlighted by benevolent leadership and moral leadership aligns with the values cherished by Gen Z employees (Nikolic, 2022). They may be more inclined to collaborate with their leaders because they feel that these leaders better understand their needs, support their growth, and consequently, have higher levels of identification with them.

Lastly, our study found traditionality moderates the relationship between paternalistic leadership and leader identification. Employees with lower traditionality exhibit a stronger relationship between paternalistic leadership and leader identification. In high traditionality contexts, traditional values can mitigate the negative impact of authoritarian leadership, leading to higher leader identification. Conversely, in low traditionality contexts, authoritarian leadership significantly affects leader identification, resulting in lower ratings. High traditionality individuals are more compliant with authority, making them less sensitive to fluctuations in leadership behavior (Hui et al., 2004a; Xiong Chen and Aryee, 2007). They tend to maintain positive relationships with leaders regardless of leadership style. On the other hand, low traditionality employees react more strongly to changes in leadership behavior, given their less adherence to traditional values.

Our study also revealed that individuals with higher traditionality start with a higher baseline level of leader identification. Although they exhibit a steeper slope of change in response to paternalistic leadership, their initial identification is generally stronger. This suggests that while high traditionality individuals naturally align with leadership, their response to changes in paternalistic leadership is less pronounced due to their familiarity with hierarchical structures (Cheng et al., 2004). Low traditionality individuals, less accustomed to traditional authority, show a more pronounced response to changes in leadership behavior, reflecting their less constrained stance toward traditional values and authority.

Taken together, high traditionality individuals’ inherent values align with paternalistic leadership, enhancing their leader identification. They are more likely to uphold traditional hierarchical norms, while low traditionality individuals, who may reject traditional values, experience a stronger impact on their leadership identification due to their more flexible stance on authority (Farh et al., 2007; Li and Ngo, 2017).

### Practical implications

In the Chinese cultural context, the three dimensions of paternalistic leadership that are most acceptable are benevolent leadership and moral leadership, both deeply resonating with people. Firstly, leaders should engage in acts of kindness and assistance toward Gen Z employees, allowing them to feel the organization’s care and warmth. This enhances employees’ sense of belonging and responsibility to the company. Providing a favorable working environment and access to information resources also encourages employees to contribute creatively to the organization. Secondly, leaders should prioritize their own conduct, establishing correct values that align with the organizational culture. As leaders, setting an example for employees, conveying positive energy, and inspiring them to actively contribute to the company’s development are crucial. Lastly, leaders should minimize autocratic and authoritative practices, avoiding a commanding tone. Instead, they should engage in communication with others in an egalitarian manner, fostering cooperation between employees and leaders.

Importantly, across all three leadership dimensions, our findings highlight the central role of leader identification as a practical lever to reduce counterproductive work behavior. It is not only the direct actions of leaders that matter, but also how these actions foster employees’ psychological connection and attachment to the leader. Strengthening leader identification serves as a key pathway through which benevolent and moral leadership reduce counterproductive tendencies, and through which authoritarian leadership can, unfortunately, amplify them. By enhancing leader identification, organizations can indirectly lower the likelihood of counterproductive actions and promote overall workplace harmony. Therefore, leadership practices that foster identification, such as demonstrating care, acting with moral integrity, and maintaining respectful, trust-based relationships, are essential strategies for improving organizational outcomes among Gen Z employees.

As Gen Z employees continue to enter the labor market, there is a notable departure in the traditional values of these new-generation workers compared to the past. This calls for adaptive and flexible responses from paternalistic leadership to address the diverse needs of different employees. On the one hand, when dealing with high traditionality employees, it is crucial to emphasize respect and humility. Leaders should highlight the importance of a respectful and humble relationship between leaders and employees, aligning with the traditional Confucian emphasis on “respect for elders and love for the young.” Cultivating an atmosphere of mutual respect can enhance the trust and identification of highly traditionality employees with leadership. Simultaneously, instilling a sense of responsibility and obligation is essential. High traditionality employees typically value duty and a sense of responsibility.

On the other hand, it is imperative to adapt flexibly to low traditionality employees. Given that low traditionality employees are more sensitive to social exchange relationships, paternalistic leadership needs to be more adaptable to their needs. In contrast to high traditionality employees, leaders can strengthen leadership identification among low traditionality employees by establishing more egalitarian and interactive communication channels. Unlike high traditionality employees, leaders can further enhance identification among low traditionality employees by establishing more egalitarian and interactive communication channels. Recognizing the heightened sensitivity of low traditionality individuals to social exchange relationships, leaders should prioritize open and transparent communication that encourages active participation and feedback. This approach not only acknowledges the distinct preferences of low traditionality employees but also fosters a collaborative and inclusive organizational culture.

### Limitations and future directions

Our study offers valuable insights into the dynamics of paternalistic leadership, leader identification, and counterproductive work behavior among Chinese Gen Z employees, making a meaningful contribution to the literature on paternalistic leadership. Nonetheless, several limitations remain, which future research should aim to address.

Firstly, all variables within this study were self-reported by Gen Z employees. While statistical analysis indicated no significant common method bias in our dataset, it is important to note that complete exclusion of such variance cannot be guaranteed. We recommend future research may opt to gather data from diverse sources, for example, the counterproductive work behavior of Gen Z employees can be evaluated by their leaders.

Secondly, this study was conducted within companies in mainland China, consequently, the generalizability of our findings may be constrained, particularly when considering variances in Western cultural contexts. In Western research, paternalistic leadership is sometimes examined as a single dimension, focusing either on benevolence or authoritarianism, each linked separately to employee outcomes (Ehrnrooth et al., 2024). By contrast, Chinese research typically adopts a three-dimensional model that integrates authoritarianism, benevolence, and morality into a unified leadership style. The effects of benevolent and moral leadership may be particularly culturally embedded, as they resonate with Confucian values and hierarchical norms that are more common in Chinese organizations. At the same time, the observed link between authoritarian leadership and counterproductive work behavior may reflect a more general psychological mechanism, such as autonomy frustration, that could extend beyond cultural boundaries. These cultural differences suggest that leadership styles effective in China may not translate directly to Western contexts. Thus, future research could replicate and compare our findings in other industries or different cultural contexts.

Finally, the results showed Gen Z employees’ leader identification played a partial mediation role in our model, which indicated there might be other mediating mechanisms in the relationship between paternalistic leadership and employees’ counterproductive work behavior. Hence, alternative mediations like emotional exhaustion (Lebrón et al., 2018), abusive supervision (Sulea et al., 2013), and work resources (Shen and Lei, 2022), could be considered from a different angle to investigate the mechanism between them in future research. We encourage future research to explore from multiple perspectives what influences Gen Z employees’ counterproductive work behavior.

## Data Availability

The raw data supporting the conclusions of this article will be made available by the authors, without undue reservation.
